# Ocular stem cells: a status update!

**DOI:** 10.1186/scrt445

**Published:** 2014-04-22

**Authors:** Kamesh Dhamodaran, Murali Subramani, Murugeswari Ponnalagu, Reshma Shetty, Debashish Das

**Affiliations:** 1 Stem Cell Research Lab, Narayana Nethralaya Foundation, Narayana Nethralaya, Narayana Health City, 258/A Bommasandra Industrial Area, Hosur Road, Bangalore 560099 Karnataka, India; 2 School of Biosciences and Technology, Vellore Institute of Technology, University of Vellore, 632014 Tamilnadu, India

## Abstract

Stem cells are unspecialized cells that have been a major focus of the field of regenerative medicine, opening new frontiers and regarded as the future of medicine. The ophthalmology branch of the medical sciences was the first to directly benefit from stem cells for regenerative treatment. The success stories of regenerative medicine in ophthalmology can be attributed to its accessibility, ease of follow-up and the eye being an immune-privileged organ. Cell-based therapies using stem cells from the ciliary body, iris and sclera are still in animal experimental stages but show potential for replacing degenerated photoreceptors. Limbal, corneal and conjunctival stem cells are still limited for use only for surface reconstruction, although they might have potential beyond this. Iris pigment epithelial, ciliary body epithelial and choroidal epithelial stem cells in laboratory studies have shown some promise for retinal or neural tissue replacement. Trabecular meshwork, orbital and sclera stem cells have properties identical to cells of mesenchymal origin but their potential has yet to be experimentally determined and validated. Retinal and retinal pigment epithelium stem cells remain the most sought out stem cells for curing retinal degenerative disorders, although treatments using them have resulted in variable outcomes. The functional aspects of the therapeutic application of lenticular stem cells are not known and need further attention. Recently, embryonic stem cell-derived retinal pigment epithelium has been used for treating patients with Stargardts disease and age-related macular degeneration. Overall, the different stem cells residing in different components of the eye have shown some success in clinical and animal studies in the field of regenerative medicine.

## Introduction

Pluripotency, the capacity to differentiate into multiple lineages, and proliferation are two characteristic attributes of stem cells. These cells are capable of replacing damaged or diseased cells under certain circumstances. Regenerative medicine or stem cell-based therapy has now reached a state where ocular tissues damaged by disease or injury can be repaired and/or regenerated. The ease of access for the therapeutic procedure as well as follow-up together with its immune-privileged status makes the eye an ideal organ for studying regenerative medicine. Such therapy involves various procedures where stem cells are injected into both the cellular and extracellular matrix microenvironments [1]. Corneal epithelial cell transplantation has been the most widely used stem cell-based therapy following bone marrow transplantation.

Stem cell-based treatment in ophthalmology follows either a cell replacement therapy strategy or a strategy involving trophic factor-based guidance cues. Throughout treatment, outcomes depend on our in-depth knowledge of the disease, the source of stem cells, the mode of treatment and the plausible mechanism driving the therapeutic outcome [2].

In this review we discuss region-specific stem cell populations and their respective functions in cell-based therapy. We also address possible hurdles to therapy and means to overcome these in our pursuit of regenerative medicine applications in the field of ophthalmology.

## Cornea (limbus and stroma)

The cornea is at the outermost surface of the eye and safeguards transparency, which is crucial for vision. The corneal stem cell population is located in the periphery of the cornea, in the limbus; these cells are termed limbal epithelial stem cells (LESCs) [3-6]. Stroma comprises 90% of the volume of the cornea and, unlike the self-renewal of epithelia, the homeostasis of stroma is not based on a cycle of cell death and mitotic renewal.

### Identification and isolation

Stem cells in the corneal epithelium are located in the basal layer of the limbal region at the corneal periphery, called the palisades of Vogt [3]. These are visualized in small clusters and are closely associated with the stromal matrix and the basement membrane, thereby assisting in cell-cell, cell-extracellular matrix and paracrine signaling communication. The corneal epithelial basal layer is composed mostly of transient amplifying cells at various stages of maturity.

LESCs are identified by their elevated expression of an isoform of the transcription factor p63 along with a high nuclear to cytoplasmic ratio [7,8]. ABCG2 (ATP binding cassette sub family G member 2) positivity has been detected in LESCs as well as several other cells residing in the suprabasal limbus and these markers have the potential to identify the LESC population based on their staining ability in clusters of progenitor-like cells in the limbus [9,10]. Reports also indicate that Musashi-1, an RNA binding protein, can be used to specifically stain LESCs [11,12]. Corneal stem cells also express enolase, cytokeratin (CK)19, and vimentin but do not express CK3, CK12, or Connexin 43, which are present in corneal epithelial cells [11,12].

Stromal multipotent clonal cells have been identified and expanded to neurospheres in cultures [13,14]. Corneal stromal stem cells are located in the anterior stroma sub-adjacent to the basal side of the palisades of Vogt [15]. Stem cells in the stroma were identified as a side population using the DNA-binding dye Hoechst 33342. These cells expressed genes encoding ABCG2, Bmi1, CD166, c-kit, Pax6, Six2 and Notch1 as well as mesenchymal stem cell and early corneal developmental markers. When differentiated, corneal stromal stem cells expressed keratocyte markers such as keratocan, ALDH3A1, CXADR, PTDGS and PDK4 [16].

### Therapeutic implications

LESC deficiency is pathological, either partially or completely, and is caused by either mechanical injury or chemical and thermal burns or acquired by diseases such as aniridia and Stevens Johnson syndrome. Treatment of such conditions involves LESC transplantation therapy. LESCs from the healthy eye in unilateral cases of ocular disease are expanded *ex vivo* for therapeutic purposes using protocols involving amniotic membrane or fibrin in the presence or absence of growth-arrested 3 T3 fibroblast feeder layers. Alternative, experimental sources for LESCs for cell-based therapy include buccal mucosal epithelial cells, hair follicle stem cells, and human embryonic stem cells (ESCs) [17,18]. Among non-limbal cell types, cultured oral mucosal cells and conjunctival epithelial cells have been transplanted to treat limbal stem cell deficiency in humans [19,20].

Recent research shows that the peripheral cornea contains a higher density of keratocyte precursors with high proliferative capacity. A three-dimensional construction using corneal keratocyte precursors and gelatin hydrogels provided cues for attracting keratocytes and extracellular matrix in scarred stroma [21]. Du and colleagues [22] demonstrated restoration of corneal transparency, stromal thickness and collagen fibril defects after injecting corneal stromal stem cells in mice. If successful, such therapy would eliminate the shortage of donor corneas needed for transplantations. Although stem cell transplantation is performed worldwide, variability in clinical outcomes implies that standardized protocols need to be established. Further validation and quality assessment studies on these cell types could provide therapeutic solutions for ocular surface reconstruction, and may also provide insights into the feasibility of their use for reconstruction of tissues beyond the ocular surface.

## Conjunctiva

The conjunctiva, apart from being a barrier to pathogenic entry, is a highly vascularized connective tissue that provides channels for proper flow of nutrients and fluids. Conjunctival cells undergo renewal similar to the corneal epithelium, but the source of the stem cells for this remains elusive [23].

### Identification and isolation

Conjunctival stem cells can differentiate into either mucin-producing goblet cells or an epithelial cell. The dividing basal cells migrate from the bulbar conjunctiva to the corneal surface and differentiate. Conjunctival epithelial cells are negative for CK3 and CK12 but positive for CK19. The stem cells residing in the fornical niche can differentiate into epithelial cells as well as goblet cells, as shown in clonal culture assays. This provides strong evidence that the stem cell population for conjunctiva renewal is in the fornix region [24,25].

### Therapeutic implications

Ocular processes that affect the cornea also affect the conjunctiva. Conjunctival scarring, cicatricial pemphigoid, thickening, dry eye or mucin deficiency are some of the conditions affecting the conjunctiva. Conjunctival autografts, oral mucous membrane grafts, nasal turbinate mucosa grafts and amniotic membrane are often used to treat conjunctival stem cell deficiency and scarring [18]. Conjunctival cells cultured on amniotic membrane have been used for cell transplantation in patients with limbal stem cell deficiency. Recent patient follow-up reports have shown that transplantation of autologous conjunctival epithelial cells improved the clinical parameters of total limbal stem cell deficiency with respect to vision acuity, impression cytology and *in vivo* confocal analysis [18,26]. These cells were cultivated *ex vivo* (on amniotic membrane) in Dulbecco's modified Eagle's medium with Ham’s 12 in the presence of epidermal growth factor, insulin, cholera toxin and hydrocortisone to derive the corneal lineage; the cells were transplanted after 2 weeks of culture. Ultrathin polymembrane (epsilon-caprolactone) substrate has also been shown to support conjunctival epithelial cell proliferation [27].

## Iris

The iris divides the space between the cornea and lens into anterior and posterior halves. The stroma and the vasculature of the iris are developed from the anterior region of the optic cup [28].

### Identification and isolation

Iris pigment epithelial cells have the ability to grow in spheres and express markers of neural stem/progenitor cells such as Nestin, Msi and Pax6. Studies from mouse iris have revealed that these cells can also be differentiated to neuronal as well as glial lineages and express markers such as Chx10, Rho, Otx2 and Olig2 [29].

### Therapeutic implications

Though the iris pigment epithelial cells have potential to be used in cell-based therapy, not much work on validation and quality assessment has been done. These cells can be transdifferentiated into retinal neuronal cells expressing retinal-specific markers [30]. Further studies are needed before iris pigment epithelial cells can be used clinically.

## Ciliary body

The ciliary body produces the aqueous humor and is involved in regulating the aqueous flow, blood flow, intra-ocular pressure and maintenance of the immune-privileged status of the anterior chamber [31].

### Identification and isolation

Ciliary body stem cells are derived from ciliary epithelium and undergo lineage-specific differentiation to retinal tissues. The ciliary-derived progenitor cell population expresses neuronal/retinal markers such as Nestin, Chx10 and Pax6. Ciliary epithelial cells can be cultured *in vitro*, forming neurospheres expressing transcription factors (Sox 2 and Pax 6) and retinal markers (Lhx2, Dach1, Six 3) [32].

### Therapeutic implications

During homeostasis ciliary epithelium maintains a balance between epithelial and neuronal cell types, whereas during disease ciliary epithelium cells can act as donor cells for retinal repair. Studies so far have revealed that ciliary epithelium cells differentiate well into the retinal lineage cells that express retinal markers but do not integrate with existing retinal architecture. Recently, Gualdoni and colleagues [33] and Yanagi and colleagues [34] reported that ciliary epithelium cells lack the potential to differentiate into photoreceptors, suggesting that the cells need to be reprogrammed to be useful as a source of new photoreceptors. Further studies are warranted so we might realize the potential of these cells in clinics. Cicero and colleagues [35] reported that, although ciliary epithelium stem cells expressed retinal markers, each cell contained pigments and had membrane interdigitations and epithelial junctions. Ballios and colleagues [36] showed that clonally derived retinal stem cell progeny from ciliary epithelium can differentiate into mature rhodopsin-positive cells using a combination of exogenous culture additives (fibroblast growth factor, heparin, retionic acid, taurine). Inoue and colleagues [37] demonstrated that modulation of the retinal transcriptional factors OTX2, CRX and CHX10 increases the potential of retinal stem cell progeny derived from the cilliary margin of adult human eye.

## Trabecular meshwork

The trabecular meshwork (TM) is a tissue between the cornea and iris in the anterior region that is responsible for drainage of aqueous fluid. The balance between aqueous secretion and outflow determines intraocular pressure, which is a risk factor for the development of glaucoma. TM cells help to remove debris in the circulating aqueous humor [38].

### Identification and isolation

TM cells express vimentin, non-muscle actin, aquaporin-1, acetylated and acetoacetylated alpha-2 adrenergic receptor, matrix GLA protein and chitinase-3-like-1 [39-41]. Recently, the isolation and characterization of TM cells have been widely studied. These studies suggest that TM cells have stem cell-like properties, expressing mesenchymal cell-associated markers such as CD73, CD90, and CD105, and the ability to differentiate into adipocytes, osteocytes, and chondrocytes [38,42]. Further, studies showed that TM stem cells isolated as a side population or as clones expressed specific stem cell markers such as ABCG2, Notch1, OCT-3/4, AnkG, and MUC1 [38]. These stem cells could differentiate into the TM lineage and expressed AQP1, CHI3L1, and TIMP3 markers and had a phagocytic function [38,42].

### Therapeutic implications

Lowering the intra-ocular pressure is an aim of treatments for glaucoma. The idea for this came primarily from the observation that TM cell division increased after argon laser trabeculoplasty [43]. Topical and oral medications, argon laser trabeculoplasty and some surgical approaches (for example, implant blebs) are current first-line treatments. A very recent study reported that stem cells isolated from human TM and expanded *in vitro* showed evidence of the ability to home to mouse TM and differentiate into TM cells *in vivo*[44]. The expanded TM stem cells expressed the stem cell markers ABCG2, Notch1, and MUC1 and were positive for expression of the TM marker protein CHI3L1. These TM cells were multipotent and had phagocytic properties [38,45]. Some groups are working on transplanting TM cells or TM progenitor cells combined with argon laser trabeculoplasty as a novel cell-based therapy for glaucoma [38,43-45].

## Lens

The lens is composed of the lens capsule, epithelium and fibers and, like the cornea, is transparent. Lens stem cells are hypothesized to reside in the lens capsule, although they have not yet been identified. It is plausible that they come from the ciliary body, which is anatomically close to the lens [46].

### Identification and isolation

Lens capsule regeneration has been shown to occur in lower vertebrates from cells residing in the ciliary body. The lens stem cells might thus reside in the lens capsule [47,48]. Lens stem cells have not yet been identified.

### Therapeutic implications

Lens progenitor cells have been derived from human ESCs as well as induced pluripotent stem cells (iPSCs) [48]. Lens stem cells are presumed to have a role in maintaining the lens transparency and might be important in cataractogenesis or other lens abnormalities

## Retina

The retina represents the connecting link between visual input and image processing in the brain. Retinal diseases mostly result in irreversible damage to the visual pathway. Several studies in animal models have achieved some amount of success using transplantation of photoreceptors, endothelial cells and retinal pigment epithelium (RPE) [17,48].

### Therapeutic implications

Most therapeutic application studies have been conducted on murine retinal disease models. Diseases in the inner retina include retinopathy (ischemic conditions) and optic neuropathy, which cause damage in the retinal ganglion cells and amacrine cells [49]. Transplantation of bone marrow-derived mesenchymal stem cells into the vitreous of a retinal ischemia mouse model demonstrated ganglion cell neuroprotection [50]. Cell transplantation in a retinal degeneration model has shown promising visual outcomes but the extent of the curative effect remained unclear [51-55]. The injected stem cells integrated into the retinal and subretinal microenvironment modulated differentiation of different cell types [51,52]. These transplanted cells integrate in a temporal-dependent manner that occurs only during rod genesis.

Clinical trials using fetal retinal cells have been conducted in patients with retinitis pigmentosa and age-related macular degeneration. In recent work, the entire retina has been replaced with differentiated stem cells rather than just single cells [53]. The three-dimensional neural retina was grown in culture from mouse ESCs [54]. Bilayer cups developed through morphogenesis of ESCs cultured with extracellular matrix. Most of the effects of transplantation seem to be based on the trophic factors used rather than a cell integration effect. Further studies and better sources of stem cells need to be investigated [55].

## Photoreceptors and retinal-pigmented epithelium

The current stem cell-based therapies for retinal diseases focus on supplementing or replacing photoreceptors and RPE in the affected retina.

### Therapeutic implications

Recently, safety and efficacy results were obtained from a clinical trial of subretinal transplantation of RPE cells derived from human ESCs. Several groups have shown the capacity of human ESCs to differentiate into RPE with variable success rates [56]. Recently, the differentiation efficacy was increased 30-fold by adding vitamin B3 and activin A protein [51]. Murine disease models such as Leber's congenital amaurosis rat have been used to study transplantation with differentiated retinal precursors; no teratoma formation was observed but the curative outcome needs to be followed up [57].

Transplantation of stem cell-, stem cell precursor- and iPSC-derived photoreceptors has resulted in functional recovery in animal models of retinal degeneration. Studies by several groups have demonstrated integration of photoreceptor precursors derived from postnatal retinas into degenerated mouse retina [52,53,58,59]. Tucker and colleagues [60] demonstrated that adult fibroblast-derived iPSCs differentiated into retinal precursor cells expressing retinal as well as photoreceptor markers (Pax6, CRX, recoverin and rhodopsin). Moreover, research has progressed from differentiating ESCs into photoreceptor lineages to determining the type of cell and day of culture required for successful transplantation [61-63]. Investigating methods to improve and support transplantation, Tucker and colleagues [64] demonstrated that a xeno-free substrate and extracellular matrix-coated dishes resulted in similar differentiation of iPSCs to retinal cells.

Cultured RPE cells were transplanted into a rat model of age-related macular degeneration generated by defective photoreceptor phagocytosis [65]. The results revealed clearing of photoreceptor debris and regaining of visual function. Clinical trials on replacement of RPE in age-related macular degeneration resulted in transient vision recovery, with an autologous source providing better results [66].

Advanced Cell Technology Inc. (Marlborough, MA, USA) is currently conducting a phase I/II clinical trial on treating macular dystrophy using human ESC-derived RPE cells [67,68]. Before RPE transplantation can be used in humans, further studies are necessary to determine how these cells can be integrated effectively into the retina without resulting in malignancy and immunogenicity [69].

## Choroid

The choroid is derived from mesoderm and neuroectoderm. Choroidal stem cells obtained from murine studies reveal mesenchymal stem cell properties, expressing markers such as Sca-1, CD90.2, CD44, CD105, CD73, ABCG2, Six2, Notch1 and Pax6. We are still far from understanding their proliferative and differentiation potential [70].

### Therapeutic implications

Choroidal and scleral cells have been differentiated into retinal lineage cells under laboratory conditions [71]. Further studies are needed to understand the biology as well as other attributes, such as differentiation and proliferative aspects, of these cells before they can be used clinically.

## Sclera

The sclera is continuous with the cornea and is composed of fibrous material with viscoelastic properties. It is responsible for maintaining ocular pressure. Scleral stem cells have a mesenchymal origin and express ABCG2, Six2, Pax6 and Notch1 [70].

### Therapeutic implications

Diseases that cause distension of the sclera, such as myopia, might be repaired using scleral stem cells. These might provide a source for bioengineering sclera for cell-based therapy [70], but more studies are needed to determine the role of scleral stem cells and treatment implications.

## Orbit

The orbit provides a scaffold for the eye. It is a bony cavity that contains the eye, optic nerve, extra-ocular muscles, nerves, fat and lacrimal gland. In recent years, adipose tissue stem cells have been studied extensively, and some research has been done on orbital adipose stem cells. Diseases such as thyroid eye disease and aggressive malignant tumors lead to neurological consequences and blindness.

### Identification and isolation

It has recently been observed that orbital fat cells contain stem cells. These are derived from neural crest cells, which are mesodermal in origin [72]. Interestingly, recent studies have isolated and characterized the orbital fat-derived stem cells. They have demonstrated that orbital adipocytes are similar to bone marrow-derived mesenchymal stem cells, sharing nearly 260 surface markers with them [73,74]. Orbit fat-derived stem cells have the potential to differentiate into osteoblasts, chondrocytes and adipocytes, and further culturing with corneal epithelial cells changed their morphology to polygonal epithelial-like cells. This was confirmed by these cells expressing the epithelial cell marker zonal-occludin-1 and differentiation markers such as CK3 and CK19 [74].

### Therapeutic implications

Destruction of corneal epithelial cells results in loss of vision. Stem cells isolated and expanded from the limbal area of the ocular surface are able to repair the corneal epithelium. However, obtaining healthy limbal stem cells and immune tolerance are still issues. Recently, Lin and colleagues [75] used orbital fat-derived stem cells to promote corneal tissue regeneration through a non-surgical route. Topical administration of fat-derived stem cells (mouse model) resulted in inhibition of inflammation and corneal re-epithelization. Therefore, orbit adipocytes are also potential candidates for cell therapy and tissue engineering of corneal epithelium. Further research into these progenitor cells may provide insight into pathological processes in orbit and other ocular damage.

## Conclusion

We provide comprehensive detail on the localization of ocular stem cells and explain the therapeutic potential of each. Ocular diseases can be classified into vascular defects, anatomical defects and neurodegenerative defects. In order to address these defects, regenerative medicine using cell replacement strategies could be highly beneficial and effective. Identification of the proper sources of stem cells is the first step towards this, followed by their isolation and characterization. Ophthalmology is the only branch of medical science that has so far gained from the field of regenerative medicine. Limbal stem cell transplantation is the only other cell-based transplantation procedure, other than bone marrow transfusion, that has been approved for patient care.

**Figure 1:**
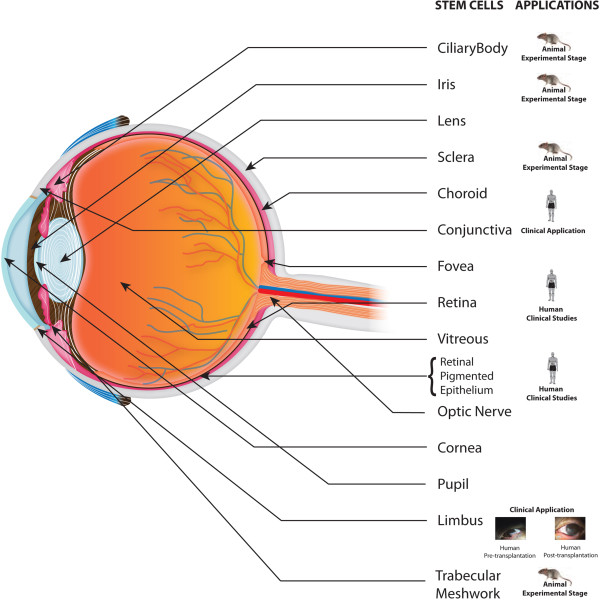
Schematic representation of sources and applications of ocular stem cells.

**Figure 2:**
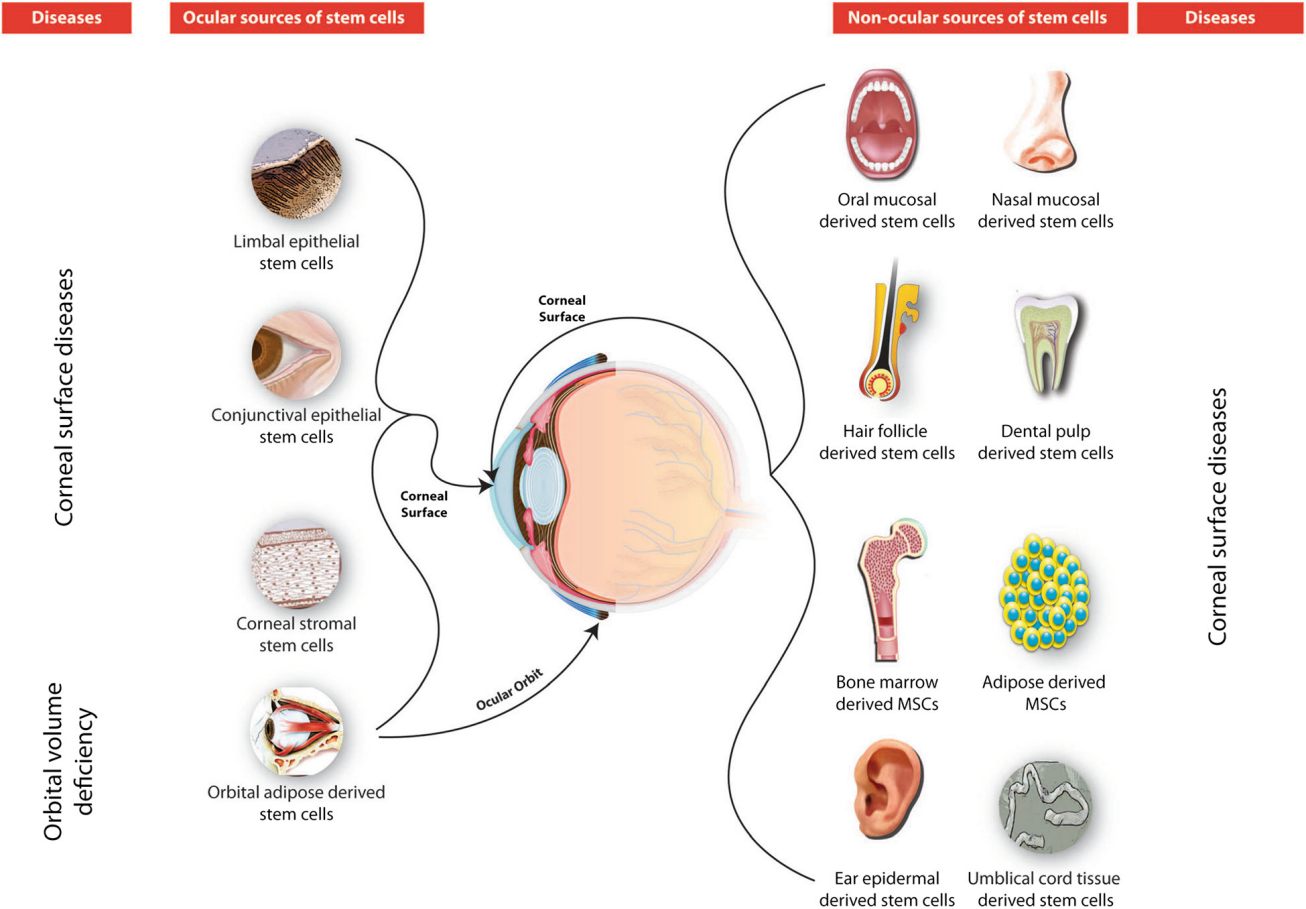
Status of ocular and non-ocular stem cell transplantation for anterior surface disorders of the eye. MSC, mesenchymal stem cell.

**Figure 3:**
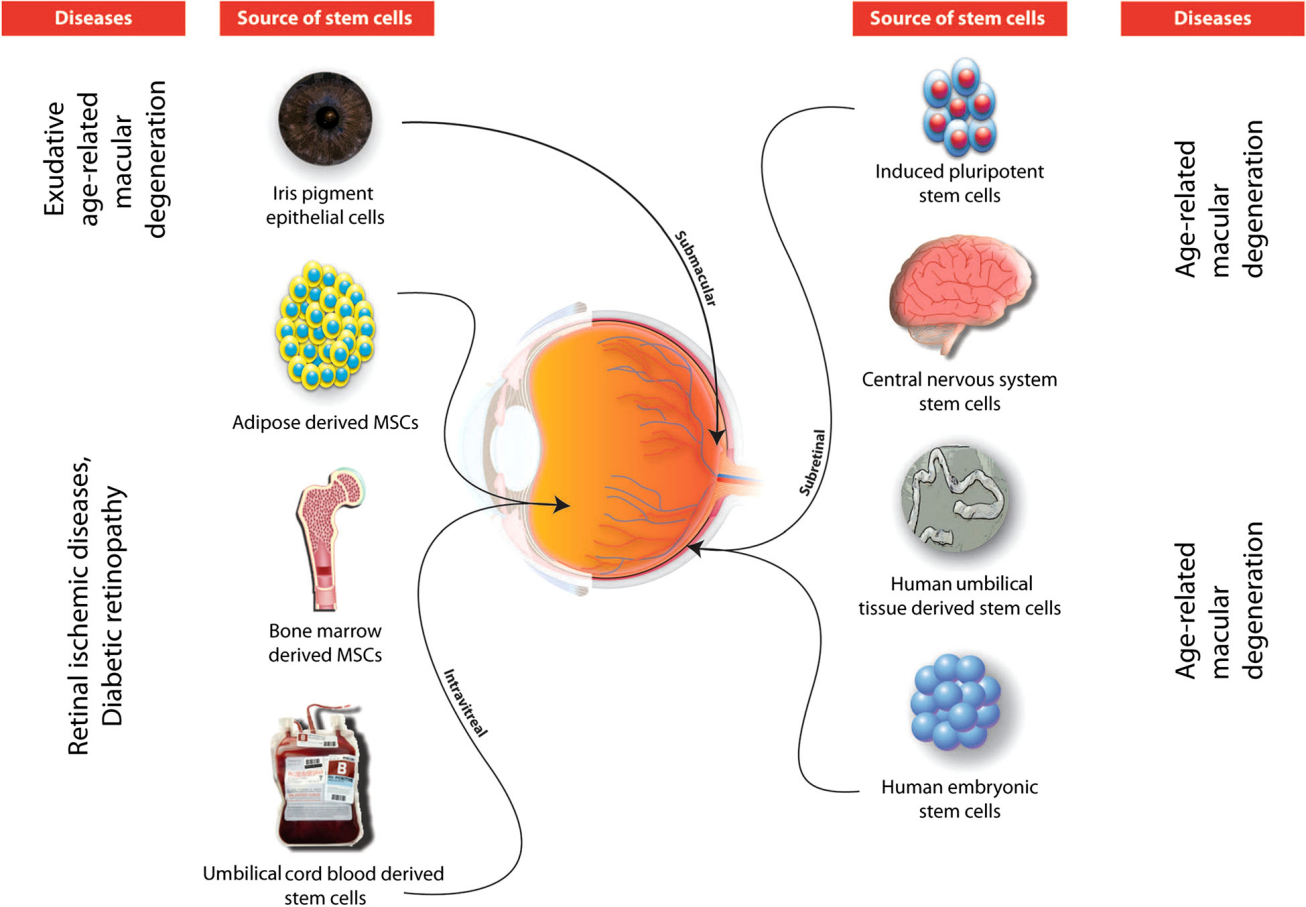
Status of ocular and non-ocular stem cell transplantation for posterior chamber disorders of the eye. MSC, mesenchymal stem cell.

Tables 1 and 2 provide summaries of the present and future prospects of stem cells for ocular therapy. Figure 1 depicts the locations of stem cells and their clinical application status. Figures 2 and 3 highlight stem cell sources used in ocular cell therapies for specific diseases. In order to harness the potential of stem cell-based therapy to provide and restore sight in blind patients, the safety of the cells needs to be studied in detail. For the successful utilization of stem cells for therapeutic purposes, small molecules can be incorporated with or conjugated to them before transplantation to promote specific differentiation pathways [76]. These cells serve to replace damaged cells and produce cytokines, growth factors, and other trophic molecules [77]. Fundamental studies are needed to unravel the roles of the Ivy league signaling pathways such as the Notch, WNT, Jak-Stat, tyrosine kinase, and Sonic hedgehog pathways. Also, alternative sources of stem cells need to be explored for their ability to integrate into the visual network. Basic researchers and ophthalmologists worldwide share optimism that stem cell therapy will in the future provide a means to restore vision.

**Table 1:** Ocular stem cells: locations, functions, markers and therapeutic development stage

**Ocular region**	**Location**	**Functions**	**Cells**	**Probable markers**	**Disease**	**Therapeutic/ experimental stage**	**References**
Cornea- limbus	Junction between cornea and conjunctiva. Basal layer of the limbal region at corneal periphery, called the palisades of Vogt	Generates transient amplifying cells that are responsible for corneal epithelial cell renewal	Limbal epithelial stem cells	Positive: isoform of p63, ABCG2, Musashi-1 Negative: CK3, CK12	Limbal stem cell deficiency	Limbal epithelial stem cell transplantation successfully used in human ocular surface reconstruction	[8,18,20]
Cornea- stroma	Corneal stromal cells are located in the anterior stroma sub-adjacent to the basal side of the palisades of Vogt	Restore organization and transparency to the cornea	Corneal stromal stem cells	Positive: ABCG2, Bmi1, CD166, c-kit, Pax6, Six2 and Notch1	Corneal scar-like disruption	Still under study	[22]
Conjunctiva	Bulbar epithelium covering slack and highly vascularized connective tissue	Proper flow of nutrients, fluids and barrier for the entry of infectious pathogens	Goblet cells, non-goblet cells, epithelial cells	Positive: keratin 19 Negative: CK3, CK12	Conjunctival scarring, cicatricial pemphigoid, thickening, dry eye or mucin deficiency	Conjunctival epithelial stem cell transplantation successfully used in human ocular surface reconstruction	[18]
Iris	Present between the cornea and lens	Control the diameter and size of the pupils	Iris pigment epithelial cells	Positive: neuronal stem/progenitor markers - Nestin, Msi , Pax6	Iritis	Potential for use in cell-based therapy and animal model studies ongoing	[30]
					Exudative age-related macular degeneration	Clinical application	[78,79]
Ciliary body	Continuation of the choroid at ora serrata	Aqueous humor production, accommodation, production and maintenance of the lens zonules	Ciliary body stem cells	Positive: neuronal stem/progenitor markers - Nestin, Chx10, Pax6, Sox2, Lhx2, Dach1, Six 3	Ciliary body detachment	Still under study	[2,31,32,34]
Trabecular meshwork	Tissue between the cornea and iris	Drainage of aqueous fluid	Endothelial cells, justacanalicular cells, mesenchymal stem cells	Positive: CD73, CD90, CD105, ABCG2, Notch1, OCT-3/4, AnkG, MUC1, AQP1, CHI3L1, TIMP3	Intra-ocular pressure	Still under study	[38,42]
Lens	Lens capsule	Play a role in maintaining the lens transparency	Lens stem cells	Not yet found	Cataractogenesis or other lens abnormalities	Still under study	[46,47]
Retina - retinal pigment epithelium	Light-sensitive layer of tissue, lining the inner surface of the eye	Visual activity	Retinal cells -RPE	Positive: Nestin, Notch 1, Chx2, Map-2, CRALBP, tyrosinase, tyrosine-related protein 1 and 2,	Retinitis pigmentosa and age-related retinal degeneration, Stargardt’s macular dystrophy and dry age-related macular degeneration	Clinical trials using transplantation of fetal retinal cells and RPE in age-related macular degeneration	[17,53,55]
Choroid	Lying between retina and sclera	Supplies oxygen and nourishment to the retina	Mesenchymal originated stem cells	Positive: Sca-1, CD90.2, CD44, CD105, CD73, ABCG2, Six2, Notch1, Pax6	Ocular neurodegenerative diseases	Still under study	[70]
Sclera	Continuation of cornea, outer layer of the eye	Maintain the shape of the eye, resistance to internal and external forces, and provides an attachment for muscles	Mesenchymal originated stem cells	Positive: ABCG2, Six2, Pax6, Notch1	Myopia	Still under study	[70]
Orbit	Scaffold for the eye	Eye protection	Orbital adipose stem cells	Positive: CD34, zonal-occludin-1, CK3, CK19	Orbital inflammatory disease, protrusion of eyeball, oribital volume deficiency	Still under study	[73-75,80]

ABCG2, ATP binding cassette sub family G member 2; CK, cytokeratin; RPE, retinal pigment epithelium.

**Table 2:** Future prospects for stem cell use for ocular cell therapy

**Cell source**	**Diseases**	**Site of inoculation/technology**	**Clinical outcome**	**References**
RPE cells	Wet AMD	Scaffolds with RPE cells into the subretinal space	*In vivo* animal studies	[81]
Human embryonic-stem-cell-derived retinal epithelium - three-dimensional culture	Retinal degenerative diseases	Autonomous formation of the optic cup (retinal primordium)	*Ex vivo* culture system	[54]
Three-dimensional culture encapsulated with retinal progenitors cells	Retinal degenerative diseases	Microfabrication processes, a novel biodegradable thin film cell encapsulation scaffold	*Ex vivo* culture system	[82]
Embryonic stem cell-derived photoreceptors	Retinal degenerative diseases	Micro-channel scaffold	Animal studies: mouse	[83]
Human RPE cells	Retinal degenerative diseases	Nanofibers	*Ex vivo* culture system	[84]
Human iPSCs	Retinal degenerative diseases	Culturing optic vesicle-like structures from human iPSCs	*Ex vivo* culture system	[85]

AMD, age-related macular degeneration; iPSC, induced pluripotent stem cell; RPE, retinal pigment epithelium.

## Abbreviations

ABCG2: ATP binding cassette sub family G member 2; CK: Cytokeratin; ESC: Embryonic stem cell; iPSC: Induced pluripotent stem cell; LESC: Limbal epithelial stem cell; RPE: Retinal pigment epithelium; TM: Trabecular meshwork.

## Competing interests

The authors declare that they have no competing interests.
